# The pendulum swings back to scoliosis screening: screening policies for early detection and treatment of idiopathic scoliosis - current concepts and recommendations

**DOI:** 10.1186/1748-7161-8-16

**Published:** 2013-10-29

**Authors:** Theodoros B Grivas, Michael Timothy Hresko, Hubert Labelle, Nigel Price, Tomasz Kotwicki, Toru Maruyama

**Affiliations:** 1 Director of the Trauma and Orthopaedic Department, “Tzanio” General Hospital of Piraeus, Piraeus, Greece; 2 Harvard Medical School, Attending Spinal Surgeon Boston Children Hospital, Boston, USA; 3 University of Montreal, Attending Spinal Surgeon, Ste-Justine Mother and Child University Hospital, Montreal, Canada; 4 University of Missouri Kansas City, Spine Section Chief, Children’s Mercy Hospital, Kansas City, USA; 5 Department of Paediatric Orthopaedics, University of Medical Sciences, Poznan, Poland; 6 Department of Orthopedic Surgery, Saitama Medical Center, Saitama Medical University, Saitama, Tokyo, Japan

## Abstract

This editorial article initiates the school scoliosis screening thematic series of the Scoliosis journal. The various issues on screening policies are discussed; clinical and practical recommendations of setting up school screening programs are also described.

## Introduction

School scoliosis screening (SSS) aims to detect back trunk asymmetry in children at risk to develop progressive scoliosis. Detection at an early stage when the deformity is likely to go unnoticed offers an opportunity for a less non- invasive method of treatment. In USA, scoliosis screening has been a major commitment of orthopaedic surgeons since the early 1960s. A large body of literature has accrued which reported a great deal of clinical experience, [1].

In Lyon, France, at the 2013 annual meeting of the Scoliosis Research Society, Dr Stuart Weinstein reported the results of the Bracing in Adolescent Idiopathic Scoliosis Trial (BrAiST). These results were simultaneously published on line in the New England Journal of Medicine, [2], picked up by the NY Times, [3], and the USA National Public Radio (NPR). The results and implications of this trial were also posted on the SRS website, [4]. The study was funded by the National Institute of Arthritis and Musculoskeletal and Skin Diseases and others; BRAIST ClinicalTrials.gov number, NCT00448448).

The reason for such energy and publicity on this issue is due to the numerous implications of the BrAIST study.

Firstly, it confirms the traditional “standard of care” for adolescent scoliosis with bracing for moderate scoliosis in growing children. Bracing is the traditional treatment which had lacked high level of scientific evidence. The BrAIST study was a randomized clinical trial with a patient preference arm – level 1 and 2 in the hierarchy of medical evidence, [5].

Secondly, the compliance data will supplement previous evidence that brace wear time is an important element of treatment that needs to be measured and assessed by patients and clinicians during any brace treatment program. All future studies comparing brace treatment regimens should include a brace monitor. For the individual patient, the brace monitor will allow the physician and patient to establish the individualized brace prescription that is necessary to maximize compliance and achieve a treatment success.

Thirdly, one of the key implications of the above mentioned report relates to scoliosis screening, beginning a new era for scoliosis screening policies. In the past, the value of a screening examination for scoliosis has been debated due in part to inconclusive evidence of the success of non-operative treatment for scoliosis. This is no longer true as the evidence from the BrAIST study established the effectiveness of bracing as early, non-operative care which can reduce the number of patients who progress to surgery, a potential cost saving for the health care system and of great benefit to patients. Due to the fact that the implementation of scoliosis school screening programs are inextricably bonded to non-operative IS treatment, it is believed that this reported “BrAiST” trial will have further impact on IS management.

## Screening policies

### History

In Europe the screening for various diseases and especially for scoliosis goes back to the first decades of the 20th century.

**Figure 1:**
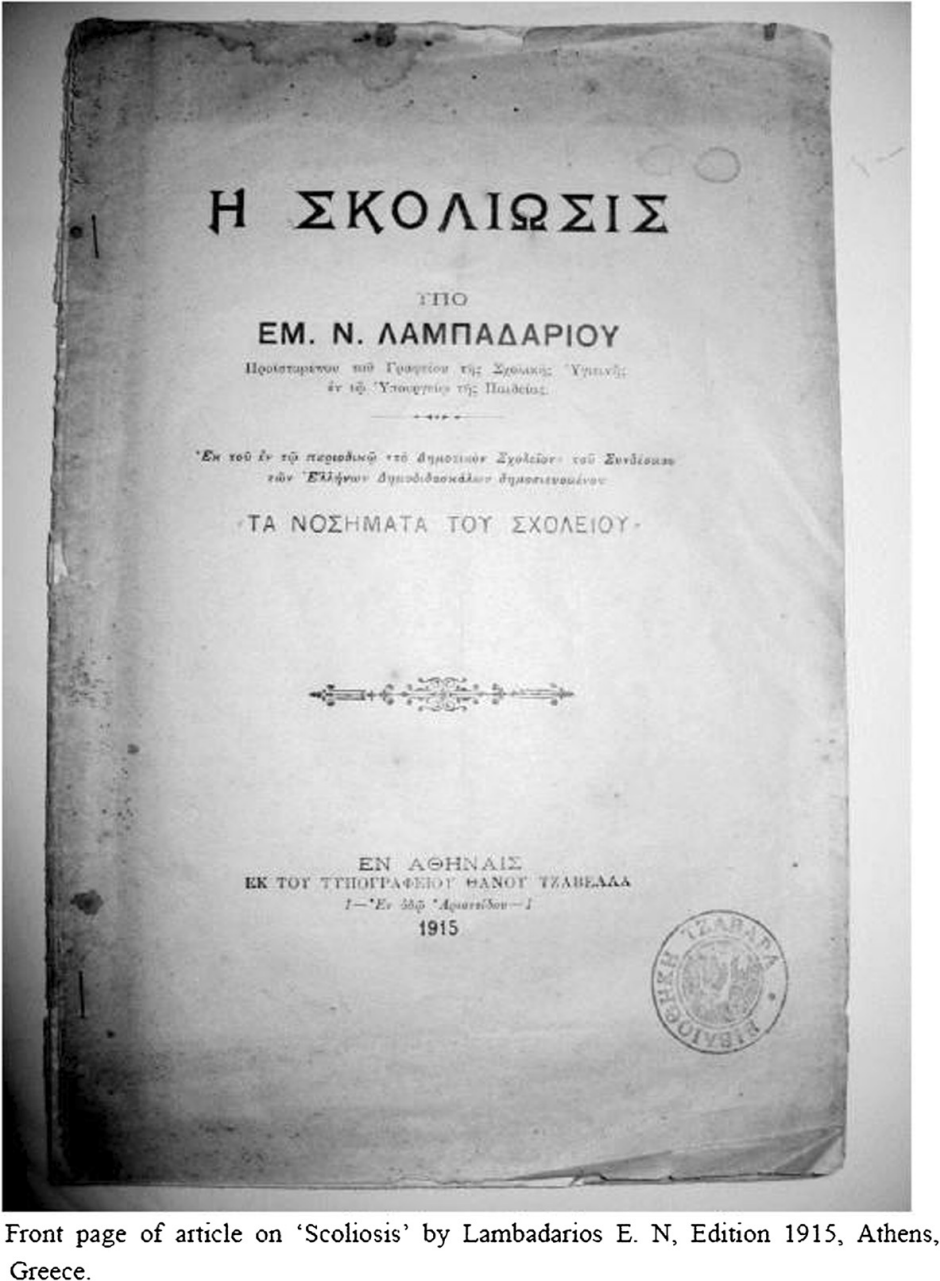
Front page of the article on “Scoliosis” by EN Lambadarios, 1915.

It is interesting to refer to a 1915 article on scoliosis published in the periodical “The Primary School”, Figure 1, and entitled “Diseases of the School” by a well-known paediatrician and specialist on school Hygiene, [6]. In this article, based on extensive personal clinical experience and compared with similar research by at least 15 international contemporary north European experts, (see Table 1), [6], the author stated that idiopathic scoliosis is a “School Disease”. Only the clinical diagnostic criteria were mentioned in the article. For the aetiology, he principally implicated inappropriately manufactured class desks, faulty sitting attitudes of children and defective class illumination. As a preventive measure he even suggested a possible cancellation of writing practice for children in the first and second primary school classes! These views prevailed for the next three decades with schoolbooks picturing faulty sitting habits etc. Girl predominance was attributed to their added sluggish home activities, [6].

**Table 1:** **Historical screening for scoliosis in Europe** (**1915**)[6]

**Author**	**Country**-**City**	**No children examined**	**Scoliosis prevalence %**	
Kotelmann	Hamburg	515	1,17	
Key	Swedeen	3219	10,4	
Combe Scoden	Lausanne	2314	24,6	
Dufestel	Paris	115	33,0	
Lambadarios	Athens	2505	20,0	
**Author**	**Scoliotics**	**Boys**	**Girls**	
Kolliker	721	144	577	
Schulthess	377	51	326	
Lambadarios	501	156%	28%	
**Author**	**Scoliotics**	**Right curve**	**Left curve**	**Double curve**
Adams	742	619	123	-
Dally	85	58	27	-
Key	751	691	60	-
Dufestel	38	27	11	-
Drachmann	130	581	727	-
Lambadaris	501	252	227	22

Owing to the presentation of newer knowledge on IS aetiology these ideas were later abandoned and forgotten.

Fifty years later, in North America, the start of screening for scoliosis began in 1963 in Aitken, a town with a population of about 10.000 in central Minnesota [1]. Dean MacEwen, MD, played an important role in the early development of school screening by implementing programs in all schools in the state of Delaware in the 1960s [1]. Consequently, the state of Minnesota pioneered spinal screening in the United States by implementing in 1973 a centrally-directed, state-wide but voluntary program, based on clinical examination [1].

### Geography of legislations on SSS policies

**Figure 2:**
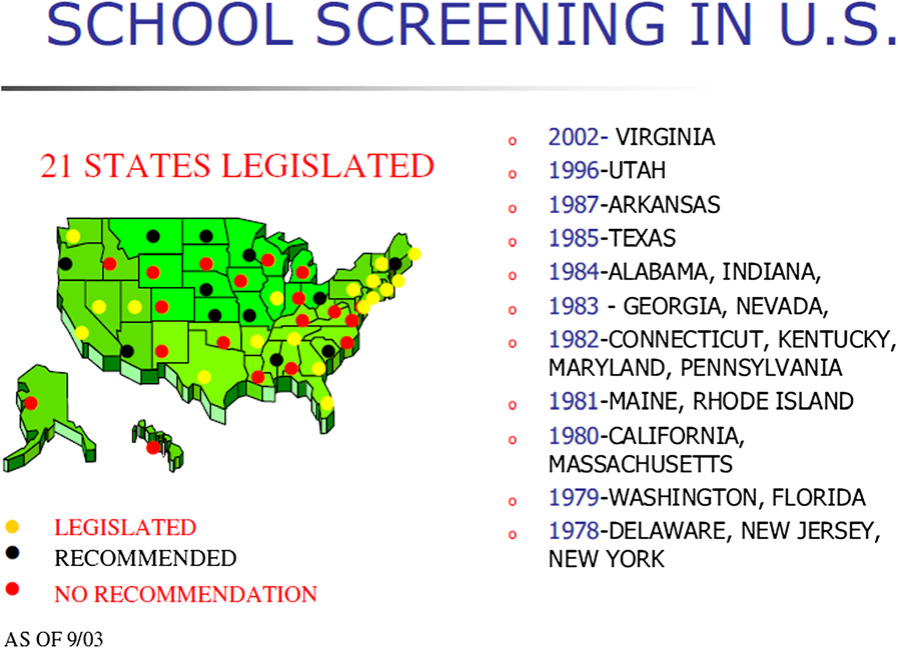
**SSS in USA.** (Courtesy Mr Joseph P. O’Brien, MBA, President & CEO National Scoliosis Foundation, Boston, USA).

Currently, less than half of the states in the United States have legislated SSS programs, and there is no nationwide requirement or standard for SSS, such policies being established at the local state, county, city, or school district level. As of 2003, 21 States had legislated school screening; 11 States recommend SSS without legislation and the remaining either have volunteer SSS or recommend against screening in schools, Figure 2. The 21 legislated States are: 2002-Virginia, 1996-Utah, 1987-Arkansas, 1985-Texas, 1984-Alabama, Indiana, 1983-Georgia, Nevada, 1982-Connecticut, Kentucky, Maryland, Pennsylvania, 1981-Maine, Rhode Island, 1980-California, Massachusetts, 1979-Washington, Florida, 1978-Delaware, New Jersey, New York [1].

In the 1970s, SSS were implemented in Canada, but were progressively discontinued a decade later based on the recommendation of the Canadian Task Force on the Periodic Health Examination.

In Japan, school-screening programs for scoliosis are mandatory by law, but each program is organized at a local level. The programs mostly are accomplished with surface topography using the moiré technique and low-dose roentgenographic techniques [1].

In Hong Kong, the school screening program is the only one in China and consists of a voluntary 3-tier assessment initiated by a University Department and has become part of the Student Health Service provided by the Department of Health since 1995.

In Singapore, routine screening of school children for spinal deformity is part of the national school health screening program since 1981.

There are no national screening programs in the other South East Asian countries, although ad hoc school screening does takes place in localized communities in Malaysia, Indonesia, and Vietnam.

In Europe there are a variety of policies and legislations for SSS. SSS is not a national policy in the United Kingdom and Poland, while in Greece, Italy, Spain, Israel, Turkey, the Netherlands and Bulgaria, it is provided on a voluntary basis, [7,8].

Similarly, school screening was introduced sporadically in Australia but by the early 1990s the cost factor led to the abandonment of most programs in government schools, with medical societies now supporting a National Self-Detection Program for Scoliosis, [1].

In 2007, school screening for scoliosis was discussed at the 4th International Conference on Conservative Management of Spinal Deformities [1], resulting in a consensus paper aimed at summarizing the current status of school screening and defining areas of consensus within 35 health professionals across the globe, [1].

In 2008, the American Academy of Orthopaedic Surgeons (AAOS), the Scoliosis Research Society (SRS), the Pediatric Orthopaedic Society of North America (POSNA), and the American Academy of Pediatrics (AAP) issued an information statement on screening in AIS, [9]. They expressed concerns that the 2004 changes in position by the United States Preventive Services Task Force (USPSTF), [10], recommending against the routine screening of asymptomatic adolescents for idiopathic scoliosis, came in the absence of any significant change in the available literature, in the absence of any change in position statements by the AAOS, SRS, POSNA, and AAP, and in the absence of any significant input from specialists who commonly care for children with scoliosis.

### Current evidence about SSS

In 2011 the Scoliosis Research Society (SRS) presidential line formed the SRS International Task Force on Scoliosis Screening, with the mandate to develop an international group of the SRS to explore Scoliosis screening from a multi-national perspective. The committee used a recent systematic review on the effectiveness of scoliosis screening, [11], and a conceptual framework of analysis used in other screening programs with success, which includes all of the World Health Organisation (WHO) criteria for a valid screening procedure. The full report of this Task Force is published in the current thematic issue, [7], and concludes that Scoliosis screening is recommended as a valuable procedure in 4 of the 5 following domains studied: technical efficacy, clinical, program and treatment effectiveness. The existing literature does not, however, provide sufficient evidence to make a statement concerning the last domain studied, cost effectiveness. This article and the above mentioned reported “BrAiST” trial, suggest that policy statements from professional organizations and governmental agencies regarding scoliosis screening will need to be re-assessed.

### The impact of SSS on frequency of surgical treatment

The earlier data reported on this issue appear to be in some way inconsistent and inconclusive. For example, in Minnesota, USA, a place with school screening in practice, a decreasing frequency of IS surgery was found, beginning in 1974 and continuing through 1979, [12]. Torell et al. [13] reported that SSS reduced the number of surgically treated IS patients. In a different report [12], data on the frequency of surgical treatment per thousand children screened for 7 or more years, were disclosed from three US states: Kansas and Virginia showed no clear trend. For Minnesota, the frequency of surgery was decreasing until 1981–82, after which it increased [12].

Some more recent European reports are more convincing on the impact of conservative treatment on the frequency of surgical treatment of IS. The incidence/prevalence of surgery can significantly be reduced where high-standard conservative treatment is available [14-16]. The implication is that effective non operative treatment must start with early detection.

### The impact of SSS discontinuation

Reports on the consequences of discontinuation of SSS programs on the referral patterns of AIS patients have been published in the literature.

A recent cross-sectional study was conducted of all patients referred for suspected adolescent idiopathic scoliosis (AIS) at an initial visit to the orthopaedic outpatient clinic of a metropolitan paediartic hospital in Canada [17]. The objective was to document the appropriateness of current referral patterns for AIS in comparison to those that prevailed before discontinuation of school screening in Canada. Of the 489 referred cases, suspected of having AIS, 206 (42%) had no significant deformity (Cobb angle <10 degrees) and could be considered as inappropriate referrals. In subjects with confirmed AIS, 91 patients (32%) were classified as late referrals with regards to brace treatment indications. The authors conclude that current referral mechanisms for AIS are leading to a suboptimal case-mix in orthopaedic ambulatory care in terms of appropriateness of referral, [17].

In Norway, Adobor et al. 2012, reported that in the absence of scoliosis screening, lay persons most often detect scoliosis. Many patients presented with a mean Cobb angle approaching the upper limit for brace treatment indications. The frequency of brace treatment has been reduced while the frequency of surgery has increased during the recent period without screening compared with the period in the past when screening was still conducted, [18].

The fact is that these reported consequences of discontinuation of SSS programs were to be expected and suggest that the epidemiological concerns which lead to their discontinuation was in reality a disaster. Prevention must be a standard policy in civilized societies with medical systems caring about people's wellbeing and not about statistics, epidemiology or only cost. We always have to remember the axiom of ancient Greeks “metron of everything is man”; in other words, the measure of appraising everything is only the human being, nothing else, [1].

### The evolving aim of SSS – the ongoing controversy over its application

The goal of SSS, as it was earlier stated, is to detect scoliosis at an early stage when the deformity is likely to go unnoticed and there is an opportunity for prevention of progressive disease by early treatment which is effective and less invasive than surgical treatment [13,19-21].

In reality, a successful SSS program identifies children with a surface deformity. It does not reveal the scoliosis per se as the surface deformity does not accurately predict the magnitude of scoliosis, especially in younger children [22]. As Bunnell characteristically states, [23], 'it has become apparent from many reports that, although there is a significant correlation between clinical deformity and radiographic measurement, the standard deviation is so high that it is not possible to reliably predict the degree of curvature from surface topography in any given patient by any technique'.

This described phenomenon is the cause of over-referrals from SSS programs generating a burden of care and adding to the ongoing controversy over the application of SSS. Therefore, it must be recognized that the goal of a SSS program is to identify the school-aged population at risk for developing scoliosis rather than to definitively diagnose the condition. There is something else that must be highlighted and clearly understood; SSS program aims at detection of surface deformity and/or the existing number of scoliosis cases. A SSS program does not aim at predicting which scoliotic curves will progress to a point that will require some type of conservative or surgical treatment. The criteria used to predict progression of a small or moderate curve are unfortunately not related to SSS programs [1].

### Recommendations for improvement of a SSS program

Grivas et al. [24,25], by reviewing the collective experience of the "Thriasio" SSS program, provide specific evidence-based recommendations for the improvement of school screening effectiveness [24,25].

SSS has to be set up on a district basis and held by a team of experienced examiners who will organize and prepare everything well in advance.

All the interested parties must be informed by distribution of informative material and lectures.

Prior to the visit of the examining group to the school, the parents must fill out a consent form and the pupils must fill out a particular form regarding their personal and demographic data.

In order to increase the predictive value of school screening, we should screen girls who live in northern countries at an older age range than those who live in the south. This recommendation is based on the fact that the regression curve of both the IS prevalence and age at menarche by geographical latitude is following a parallel course, especially in latitudes northern than 25 degrees; this means that in northern latitudes, girls experience menarche at a later age and have a higher prevalence of IS. IS almost always occurs during the time of peak growth velocity, typically during the year just prior to menarche, [26].

The referral process after the screening must be standardized according to a specific protocol by documenting the positive findings of a detected curve.

As a second stage of screening, demographic and clinical parameters, including the gender, the chronological age, the age at menarche, the pattern and the magnitude of asymmetry and the growth potential must be recorded, in order for the more experienced Orthopaedic surgeons to determine whether it is necessary to x-ray a referred child or not. Approximately 25% of younger referred girls (aged <13 years old) with an ATR ≥ 7° were found to have either a straight spine or a spinal curve under 10°. In this age group the correlation between clinical deformity and radiographic measurement is poor, while in older referred girls (aged 14–18 years old) the correlation is better. Therefore, all the younger individuals who are identified with a surface deformity but without a severe scoliotic curve are at risk for IS development and need continued assessment until puberty [22].

It is crucial for everyone who participates to fully recognize the voluntary basis of the program, in order to reduce the financial cost. The financial cost can be either direct or indirect. There is no general consensus among economists as to what constitutes the indirect cost in a cost-effectiveness analysis. Indirect cost cannot be measured precisely, as it is related to the effectiveness of the school screening program. A more effective screening program has lower indirect cost. Therefore, the economic information on screening for scoliosis which is available to decision-makers should mainly be based on studies of the direct cost of such programs. Grivas et al have shown that the direct cost of a screening program can be reduced to a minimum, if it is well organized and performed on a voluntary basis [27,28].

### More benefits of SSS: Its contribution to clinical research on IS aetiology

SSS should be adopted by policy makers, because its scope goes beyond the identification of IS at an early stage contributing significantly into the research for IS aetiology.

There are pertinent reports on IS prevalence based in SSS studies that estimate the number of children who will require conservative or surgical treatment. Similarly studies originated from school screening data identified a difference in a prevalence of IS reported in blind females, the role of different age at menarche of IS and non scoliotic peers; the role of melatonin in the pathogenesis of IS, the relation between menarche and laterality of scoliotic curves.

Studies in SSS resulted in implications on the brain function, the morphology of the thorax, and its relation to the aetiology of IS. Applications of these studies are now used for the assessment of the outcomes of the surgical and non-operative treatment on the rib-cage deformity, (see DRCS and the RI).

Studies in school screening referrals also facilitated the study of the role of the lateral spinal profile in the aetiology of IS, the role of intervertebral disc in IS pathogenesis. Also gave evidence on the IS and cavus foot relationship.

Somatometric data resulted from the study of school screening data were also gathered.

The study of the parental age at birth implicated a possible epigenetic mechanism on the truncal asymmetry of a child. Similarly the study of Body Mass Index in relation to truncal asymmetry of healthy adolescents, created a physiopathogenetic concept which seems to be in common with idiopathic scoliosis.

The contribution of the resulted studies from the data gathered from SSS on IS aetiology were earlier published [29-51].

## Setting up SSS programs: clinical and practical considerations

In several spinal centers in USA and in Europe offer training for the interested practitioners on the way one could institute, organize and perform a regional scoliosis school-screening (SSS) program.

It must be highlighted that the orthopaedic community must encourage the health authorities and the pertinent institutes on the understanding of the value of SSS in every country, and the discussion of the opportunities and barriers to the development of a common protocol for SSS. Such an endeavor was recently undertaken at an educational event at Warsaw, Poland, on 27-09-2013 [52].

In order to establish a SSS program, SSS practitioners must be identified and trained to visit schools and collect, process and analyze the SSS data. Moreover, the training program must be familiarized with:

• The method to obtain the permission of the a) Administration of the Hospital and b) the Ministry of Education to perform the examination of children at schools.

• The organization of the group of examiners, comprising orthopaedic surgeons, health visitors, physiotherapists and school nurses where available.

• The items on which the team must be appropriately trained for the implementation of the school-screening process.

• Methods for communicating with the School Administration, which is significant for the success of the screening program.

• Management of the “materials” including the various forms to fill (protocols, consents)

• How to examine the children and use the relevant instruments, including the scoliometer, a body weight scale and a height measure.

• Decision making for the time and the frequency of the performance of screening, which is variable depending on many parameters (for example the proper day and program time period in order to minimally disturb the educational program of the children).

• Management of data collection and its processing and analysis.

• How children are monitored (those asymmetric children at risk of developing scoliosis or scoliotics), including referral to the outpatient clinic for re-assessment and the commencement of early non-operative treatment.

• Also, the possibility of increasing the research community’s insight into scoliosis by presenting and publicizing data collected through SSS programs.

The education of any interesting health care provider is also broadened by visiting an experienced spinal center on the SSS, and attending specialized seminars focused on the theoretical and practical issues for the introduction and implementing SSS programs.

There are countries like Greece, where the diminishing number of scoliotic curves referred for surgery has been attributed to the efficient screening programs currently employed and the availability of early non-operative treatment.

In conclusion, the authors of this editorial believe that recent evidence supports the value of scoliosis screening and that a re-appraisal of SSS and other scoliosis screening procedures is warranted.

## Competing interests

The authors declare that they have no competing interests.

## Authors’ contributions

TBG initiated the idea for a Thematic Series on the school scoliosis screening and drafted the editorial. MTH, HL, NP, TK and TM contributed their professional skills, reviewed the editorial and partially contributed in drafting of the manuscript adding certain references. MTH, HL, TK contributed by text editing. All authors have read and approved the final manuscript.
